# Imported *Plasmodium falciparum* and locally transmitted *Plasmodium vivax*: cross-border malaria transmission scenario in northwestern Thailand

**DOI:** 10.1186/s12936-017-1900-2

**Published:** 2017-06-21

**Authors:** Patchara Sriwichai, Stephan Karl, Yudthana Samung, Kirakorn Kiattibutr, Jeeraphat Sirichaisinthop, Ivo Mueller, Liwang Cui, Jetsumon Sattabongkot

**Affiliations:** 1 Department of Medical Entomology, Faculty of Tropical Medicine, Mahidol University, Bangkok, Thailand; 2 Population Health and Immunity Division, Walter and Eliza Hall Institute of Medical Research, Melbourne, Australia; 3 Mahidol Vivax Research Unit, Faculty of Tropical Medicine, Mahidol University, Bangkok, Thailand; 4 Bureau of Vector Borne Diseases, Pra Phuttabhat, Saraburi, Thailand; 5 Department of Entomology, Pennsylvania State University, University Park, PA USA

**Keywords:** Malaria transmission, Border malaria, Migration, Mosquito infection, Thailand

## Abstract

**Background:**

Cross-border malaria transmission is an important problem for national malaria control programmes. The epidemiology of cross-border malaria is further complicated in areas where *Plasmodium falciparum* and *Plasmodium vivax* are both endemic. By combining passive case detection data with entomological data, a transmission scenario on the northwestern Thai–Myanmar border where *P. falciparum* is likely driven by importation was described, whereas *P. vivax* is also locally transmitted. This study highlights the differences in the level of control required to eliminate *P. falciparum* and *P. vivax* from the same region.

**Methods:**

Malaria case data were collected from malaria clinics in Suan Oi village, Tak Province, Thailand between 2011 and 2014. Infections were diagnosed by light microscopy. Demographic data, including migrant status, were correlated with concomitantly collected entomology data from 1330 mosquito trap nights using logistic regression. Malaria infection in the captured mosquitoes was detected by ELISA.

**Results:**

Recent migrants were almost four times more likely to be infected with *P. falciparum* compared with Thai patients (OR 3.84, p < 0.001) and cases were significantly associated with seasonal migration. However, *P. falciparum* infection was not associated with the *Anopheles* mosquito capture rates, suggesting predominantly imported infections. In contrast, recent migrants were equally likely to present with *P. vivax* as mid-term migrants. Both migrant groups were twice as likely to be infected with *P. vivax* in comparison to the resident Thai population (OR 1.96, p < 0.001 and OR 1.94, p < 0.001, respectively). *Plasmodium vivax* cases were strongly correlated with age and local capture rates of two major vector species *Anopheles minimus* and *Anopheles maculatus* (OR 1.23, p = 0.020 and OR 1.33, p = 0.046, respectively), suggesting that a high level of local transmission might be causing these infections.

**Conclusions:**

On the Thai–Myanmar border, *P. falciparum* infections occur mostly in the recent migrant population with a seasonality reflecting that of agricultural activity, rather than that of the local mosquito population. This suggests that *P. falciparum* was mostly imported. In contrast, *P. vivax* cases were significantly associated with mosquito capture rates and less with migrant status, indicating local transmission. This highlights the different timelines and requirements for *P. falciparum* and *P. vivax* elimination in the same region and underlines the importance of multinational, cross-border malaria control.

**Electronic supplementary material:**

The online version of this article (doi:10.1186/s12936-017-1900-2) contains supplementary material, which is available to authorized users.

## Background

Malaria epidemiology in the Greater Mekong Sub-region (GMS) is complex, with all five parasite species present and a large variety of mosquito vectors [1, 2]. Malaria cases have been reduced to near elimination in some parts of the region, while others still exhibit high incidence, especially remote, forested areas [3, 4]. The GMS has a history as a focus for the development of anti-malarial drug resistance [5]. Political borders often also separate areas of high and low transmission in the GMS. Traditional cross-border migration in search of work, but also displacement due to population upheavals, facilitate the importation of malaria into low-transmission areas and this represents a major challenge to malaria control in this region.

Approximately three-quarters of reported cases in the GMS occur in Myanmar while the incidence rate in Thailand is low and further decreasing (average number of cases per 1000 population was 0.55, 0.48 and 0.24 in 2013, 2014 and 2015, respectively). Tak Province represents a malaria hotspot in Thailand with an estimated 6.3 cases per 1000 population in 2015 [6]. Especially, the northwestern Thai–Myanmar border represents malaria transmission hotspots and ports with high number of cases imported into Thailand. Away from the border towards the central part of Thailand, malaria incidence decreases rapidly. The usual malaria transmission pattern in western Thailand such as Tak Province, exhibits two peaks, one at the beginning of the rainy season and the other at the end of the rainy season [7]. The border is very porous and populations of seasonal labourers and refugees can move across it relatively freely. The large degree of population movement, rapid ecological changes and complex vector population dynamics make this region one of the most important transmission areas in the GMS [4, 8–12]. There is a strong case to develop harmonized cross-border malaria surveillance and control programmes in conjunction with national strategic plans in order to control cross-border malaria transmission [8–14]. This should include active and passive case detection, as well as entomological surveillance [15].

Previous studies have rarely integrated entomological with epidemiological surveillance [16–20]. Entomological surveillance provides additional information to distinguish between locally transmitted and imported malaria. A previous study in this area reported that approximately 50% of malaria patients presenting to malaria posts were of Thai nationality while approximately 29% were migrants from Myanmar [21]. Malaria infection was shown to be approximately eight times higher in ethnic Karen but whether this is due to local transmission or importation remains unclear [19]. In addition, a recent study has reported an increasing incidence of *Plasmodium vivax* in this area [6]. Importation of malaria is likely facilitated by a large proportion of asymptomatic migrants who are unlikely to be detected by routine passive case detection [22]. Furthermore, it is likely that the two main malaria species, *Plasmodium falciparum* and *P. vivax*, may exhibit very different dynamics in cross-border transmission scenarios. *Plasmodium falciparum* is much more dependent on a sustained local mosquito population, whereas *P. vivax* can endure lower mosquito numbers and highly seasonal mosquito population dynamics [23]. It is, therefore, likely that *P. falciparum* is eliminated first, but local *P. vivax* transmission is sustained for an extended period of time [24]. Vector ecology in the study area is very complex. *Anopheles minimus* and *Anopheles maculatus* are the main malaria vectors constituting >85% of the captured vector population over the course of a year (2012). However, a variety of minor vectors, including *Anopheles dirus, Anopheles annularis* and *Anopheles barbirostris* are also present and have been shown to contribute to malaria transmission [25, 26]. The present study, conducted in a border village (Suan Oi) in Tha Song Yang district, Thailand, aimed to correlate mosquito capture rates with infection and patients’ demographic data collected alongside the mosquito surveillance in order to better understand this complex transmission environment.

## Methods

### Study site

**Fig. 1:**
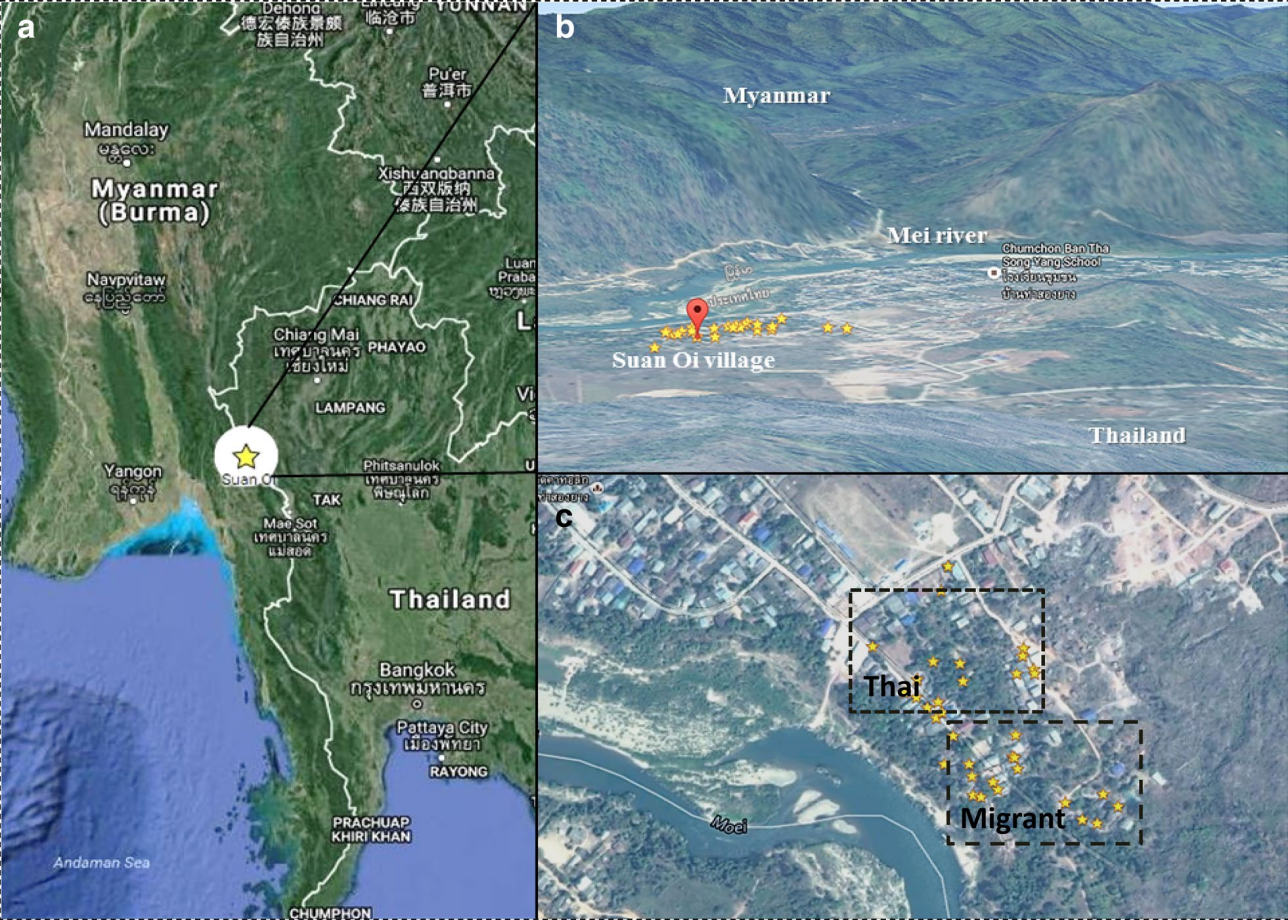
Map of the study area. **A** shows the location of Suan Oi village, Tha Song Yang district, Tak Province on the Thai Myanmar Border. **B** shows a panorama view of the area, looking west across the border into Myanmar. **C** shows an aerial view of the village with stars representing the area of CDC light trap placement from August 2011 to April 2013. **C** also shows the approximate areas predominantly occupied by Thai nationals and migrant populations, respectively. Map was modified from Google maps

The study was carried out in Suan Oi village located in Tha Song Yang district in northwestern Thailand (Fig. 1). This Thai village, with ~500 inhabitants and 290 households, borders the Kayin State of Myanmar across the Moei River. One of the 18 migration checkpoints on the Thai–Myanmar border is located in this village. In 2013, 2264 migrants from Myanmar were recorded at this checkpoint. Most of them came from different states or divisions of Myanmar (Kayin, Kayah, Taninthayi, Bago, Mon). Figure 1C shows the areas in the village that are predominantly occupied by either migrants or local Thai nationals. As one of the measures for malaria control per the Ministry of Public Health policy, the national malaria control programme conducts vector control in all active transmission areas, including the area of this study. This policy recommends that in-house residual spraying is conducted twice a year in perennial transmission areas, and annually in periodic transmission areas covering the transmission season. In addition, permethrin insecticide-treated nets (ITNs) are distributed in high transmission areas free of charge. Thermal fogging is applied during malaria outbreaks once a week for 4 consecutive weeks. Despite these control efforts, surprisingly low ITN usage was found in selected houses in Suan Oi: only 50% Thai houses and 30% migrant households were reported to use ITNs when asked, before setting the traps.

### Entomological study

Adult mosquitoes were collected using CDC light traps (BiQuip model 2836BQ, with a 6-volt battery, USA). Traps were placed in or near randomly selected households to approximately cover the extent of the village area. Traps were placed indoors and outdoors from 18.00 to 06.00 h for five consecutive nights per month in a total of 45 locations. There were a total of 266 trap placements (1330 trap nights) from August 2011 to April 2013 with the majority of trap placements in 2012. During some months, no traps were set up due to flooding and political unrest. Captured mosquitoes were transported to and sorted in the laboratory of the Medical Entomology Department, Faculty of Tropical Medicine, Mahidol University, Bangkok. Mosquito species were identified based on morphological characteristics [27]. The heads/thoraxes and abdomen of female anopheline mosquitoes were pooled and examined either individually or in pools of maximum of ten mosquitoes collected at the same time and in the same trap location. The collected mosquitoes were kept at −20 °C and ELISA assays were performed, as previously described, to detect circumsporozoite (CS) proteins of *P. falciparum* and *P. vivax* [28].

### Malaria cases

Malaria patient data were available between August 2011 and December 2014 from the Suan Oi malaria clinic. However, in the present study only the data collected in the 12 months of concomitant mosquito collection were utilized. Malaria was diagnosed by light microscopic examination of Giemsa-stained thick and thin blood smears. If slides were positive, parasite densities were determined by counting a minimum of 1000 and a maximum of 2000 leucocytes. Apart from clinical symptoms, basic demographic information such as age, gender, occupation, nationality, and migration status was also collected. Patients were divided into three groups: Thai nationality (M0), migrants residing in Thailand for 6 months or longer (M1), and migrants who stated that they had been living in Thailand for fewer than 6 months (M2). Since the ethnic background in this region does not allow for determination of nationality, the grouping was done according to The Bureau of Vector Borne Diseases (BVBD) in Thailand. Most M1 migrants are registered with the Ministry of Labour (MoL), which grants permission to stay in Thailand for a period of typically 1–2 years. People in the M2 group are often highly mobile, and are less likely to have registered with the MoL. The provincial government gives permits at the border crossings for entering that district of Thailand for 1–7 days, which can be extended by returning to the border crossing for re-authorization.

### Ethics approvals

The study was approved by the Ethics Committee of the Department of Disease Control Ministry of Public Health, Thailand and the Human Research Ethics Committees of Mahidol University and Pennsylvania State University.

### Data analysis

Logistic regression was used to test for an association of *P. falciparum* and *P. vivax* positivity (as the dependent variable), with the predictors such as age, gender, migrant status, and the monthly *An. minimus* and *An. maculatus* capture rates. The mosquito capture rate is defined as the number of mosquitoes trapped per trap and per night. The proportion of malaria-positive patients in each migrant group was plotted over the proportion of all malaria-positive patients to obtain a measure of the contribution of each migrant group to the overall malaria cases. Linear regression was used to correlate overall patient proportions positive for either *P. falciparum* or *P. vivax* with the mosquito capture rates. Statistical analyses were conducted in Stata 12 (StataCorp, USA).

## Results

**Fig. 2:**
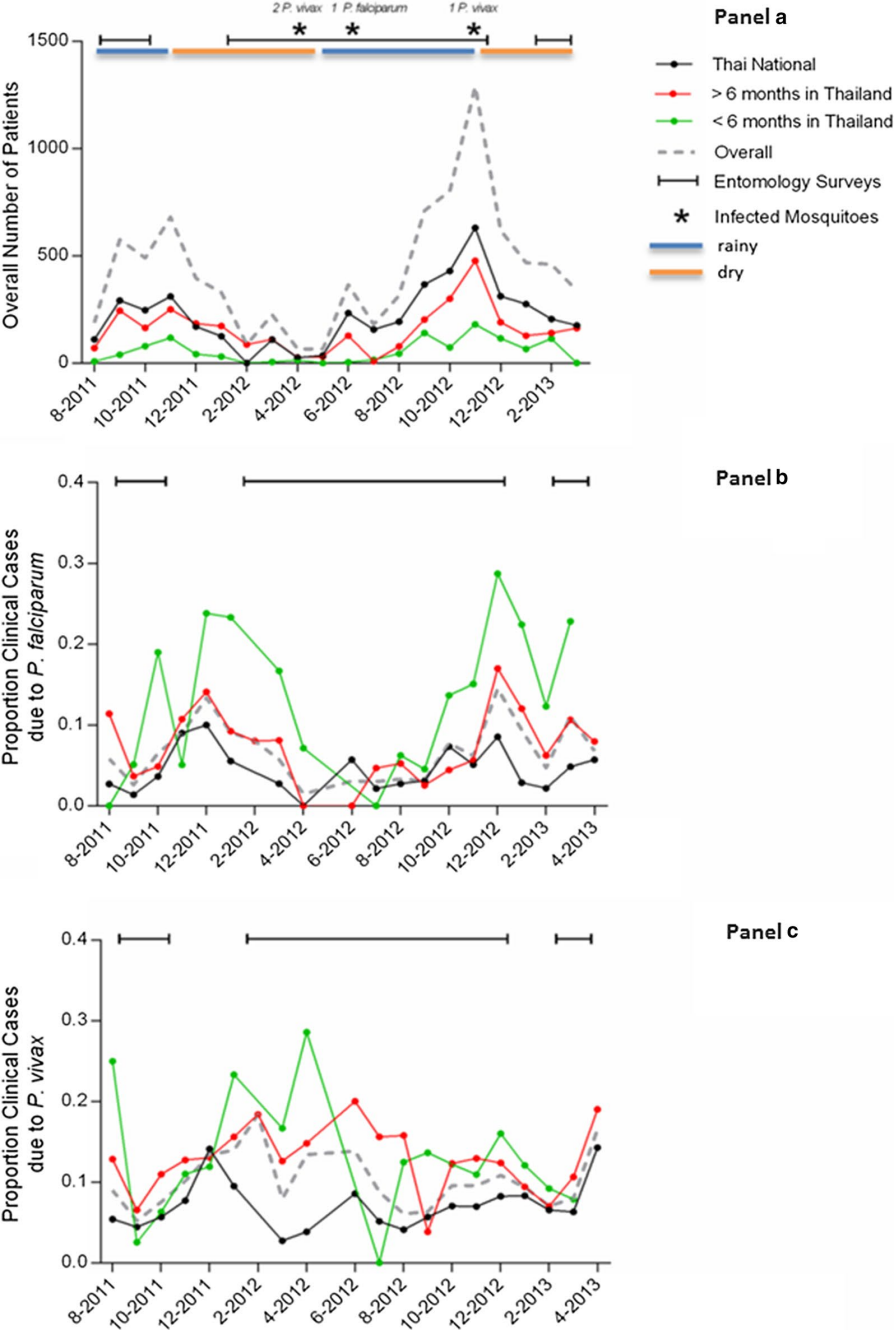
Malaria cases and mosquito capture periods in the study area. **a** shows the total malaria cases classified by population group (: overall, : Thai citizenship, : Foreigners residing in Thailand for >6 months or longer (M1), : Foreigners residing in Thailand for <6 months (M2), : entomological survey, dry/rainy season). **b**, **c** show the proportion of positive clinical cases in all patient groups over the total patients for *P. falciparum* (**b**) and *P. vivax* (**c**) infections

There were 4225 patient visits to the malaria clinic in the 12 months when mosquito trapping was conducted. The overall patient characteristics are shown in Table 1. Thirty-seven percent of the patients presented with fever (>37.5 °C). The patient population consisted of 65% Thai (M0), 33% M1 foreigners and 12% M2 migrants. About 47% of patients were male and the median age was 13 years. Microscopy diagnosed 267 *P. falciparum* and 354 *P. vivax* infections. In the same time, a total of 512 *An. minimus* and 286 *An. maculatus* were trapped in the 266 trap placements (1330 trap nights), constituting ~85% of the trapped anopheline population. Average number of mosquitoes monthly captured per trap was shown in Figure S1 (Additional file 1). Figure 2 shows the monthly malaria case numbers (Panel a) and the relative proportions of cases positive for *P. falciparum* (Panel b) and *P. vivax* (Panel c) for the different migration strata (Thai, M1, M2) during 2011–2013, number of *P. vivax* and *P. falciparum* cases per month were shown in Figure S2 (Additoinal file 2). The patient characteristics and occupation distribution per population strata were summarized in Table S1–S4 (Additional file 3). Cases numbers peaked during the transition period from rainy to dry season in both years (October–December). All population groups showed a rise in the absolute number of patients (from fewer than 50 in May/June to more than 1250 in November). However the proportion of *P. falciparum* cases in M2 migrants rose from near 0 to 20–30% in both years, whereas that for Thai nationals remained more stable (0–10%, mostly around 5%) throughout the study period. Most mosquito traps were placed in 2012. Altogether four infected mosquitoes were captured (three with *P. vivax* and one with *P. falciparum*). However, these low numbers precluded the use of sporozoite rates in the present statistical analyses.

**Table 1:** The characteristics of the patients who visited the Suan Oi malaria clinic in the study period

Patient characteristics (n = 4425)	Number (% or median range)
Age (years)	13 (0–89)
Temperature	37.0 (34.5–38.0)
Male	2080 (47%)
Fever (>37.5 °C)	1637 (37%)
Population group	
Foreigners <6 months (M2)	522 (12%)
Foreigners >6 months or longer (M1)	1480 (33%)
Thai nationality	2423 (65%)
Malaria infection	
*P. vivax*	359 (8%)
*P. falciparum*	247 (6%)
*P. vivax* with fever	126 (36%)
*P. falciparum* with fever	63 (24%)

Recent migrants (M2) contributed the highest proportion of *P. falciparum* infections and were almost four times more likely to present with *P. falciparum* (odds ratio (OR) 3.84, 95% CI 2.76–5.33, p < 0.001) in comparison with the Thai patients (M0) (Table 2). In addition, recent migrants (M2) were also significantly more likely to present with *P. falciparum* compared with long-term migrants (M1) (OR 2.49 95% CI 1.33–4.72). This suggests that *P. falciparum* infections were mostly imported. Long-term migrants were only slightly more likely to present with *P. falciparum* in comparison with Thai patients (OR 1.54, 95% CI 1.13–2.08, p = 0.006, Table 2). The contribution of the recent migrants to *P. falciparum* infections was confirmed by the high proportion of two peaks in the same periods of dry to hot season (December–March) in Fig. 2b, coinciding with the influx of migrant workers for the agricultural harvest season [19]. *Plasmodium falciparum* infections were also higher in the rainy season (August) (Fig. 2b). Importantly, *P. falciparum* positivity was not associated with mosquito capture rate for either *An. minimus* or *An. maculatus* (OR 1.15, 95% CI 0.93–1.42, p = 0.191 and OR 0.69, 95% CI 0.37–1.31, p = 0.259, respectively), further indicating that the majority of *P. falciparum* infections might be imported. Age was not significantly correlated with *P. falciparum* positivity (OR 1.00, 95% CI 1.00–1.00, p = 0.964). Males were significantly more likely to present with detectable *P. falciparum* (OR 1.71, 95% CI 1.30–2.24, p < 0.001, Table 2), which may be related to higher occupational exposure. However, in the present study no association with occupation recorded from the patient interviews was evident.

**Table 2:** Logistic regression model for *Plasmodium vivax* and *Plasmodium falciparum* infection

*P. vivax* infection	OR	z	p	95% CI	
*An. minimus* capture rate (per trap night)	1.23	2.32	0.020	1.03	1.46
*An. maculatus* capture rate	1.33	1.99	0.046	1.00	1.76
In-migration (reference: Thai nationality)					
Foreigners >6 months in Thailand (M1)	1.96	5.58	<0.001	1.55	2.49
Foreigners <6 months in Thailand (M2)	1.94	3.97	<0.001	1.40	2.70
Age (per year)	0.98	4.23	<0.001	0.98	0.99
Male	1.07	0.65	0.518	0.86	1.34

*P. falciparum* infection	OR	z	p	95% CI	
*An. minimus* capture rate	1.15	1.31	0.191	0.93	1.42
*An. maculatus* capture rate	0.69	1.13	0.259	0.37	1.31
In-migration (reference: local Thai nationality)					
Foreigners >6 months in Thailand (M1)	1.54	2.76	0.006	1.13	2.08
Foreigners <6 months in Thailand (M2)	3.84	8.02	<0.001	2.76	5.33
Age (per year)	1.00	0.04	0.964	1.00	1.00
Male	1.71	3.85	<0.001	1.30	2.24

*OR* odds ratio, *CI 95%* confidence interval of odds ratio

The overall proportion of *P. vivax* cases was relatively constant throughout the years. Both, M1 and M2 foreigners were equally more likely to present with *P. vivax* infection when compared with the Thai population group (aOR 1.96, p < 0.001, 95% CI 1.55–2.49, and aOR 1.94, p < 0.001, 95% CI 1.40–2.70, respectively). However, Thai patients presented a stable contribution to the caseload throughout the year 2011–2013, whereas the contribution of the recent migrants fluctuated and normally peaked in the dry and hot seasons of January and April 2012, coinciding with seasons of increased agricultural activity. Yet, the seasonal peaks in the migrant populations were less pronounced in year 2013 (Fig. 2c). This suggests that a significant proportion of *P. vivax* cases were acquired locally. As shown in previous studies, *P. vivax* infection was associated with age (aOR 0.98 per year of age, p < 0.01, 95% CI 0.98–0.99 per year of age).

**Fig. 3:**
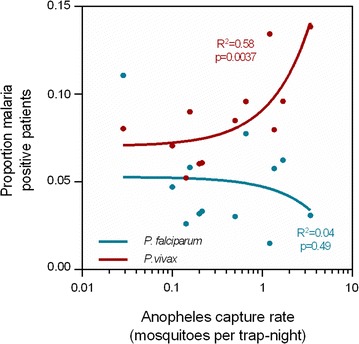
Association between combined *Anopheles minimus* and *Anopheles maculatus* capture rate (in mosquitoes per trap-night) and the proportion of malaria-positive patients. There is a strong association of mosquito capture rate with *P. vivax* cases (p = 0.0037), whereas there is no correlation with *P. falciparum* cases (p = 0.49)

The association between monthly mosquito capture rates (combined for *An. minimus* and *An. maculatus*, in mosquitoes per trap-year) and the proportion of malaria-positive cases was determined for both parasites. Consistent with local *P. vivax* transmission, *P. vivax* slide positivity was more likely in months with high *An. minimus* and *An. maculatus* capture rates (aOR 1.23, p = 0.020, 95% CI 1.03–1.46 and aOR 1.33, p = 0.046, 95% CI 1.00–1.76, respectively, Table 2). While the proportion of *P. vivax*-positive patients was significantly associated with the mosquito capture rate (coefficient of determination: 0.58, p < 0.0037), there was no association between the proportion of *P. falciparum*-positive patients and the mosquito capture rate (Fig. 3).

## Discussion

While several studies have investigated malaria risk factors in western Thailand and on the Thai–Myanmar border [29–31], entomology data were rarely incorporated into these studies [32]. The present study aimed to combine longitudinal entomological surveillance data collected using CDC light traps with passive case detection data from a local malaria clinic to gain further insights into malaria transmission dynamics in a rural setting on the Thai–Myanmar border.

The study area is one of Thailand’s most malaria-endemic regions. Tak Province in 2015 had an estimated annual incidence of 6.3 per 1000 people, approximately 26 times the national average, and ~65% of the infections were caused by *P. vivax* [6]. The area is also characterized by significant cross-border migration and migrant populations serve as an important reservoir for malaria transmission in Thailand [10]. Generally, migrant workers from Myanmar represent the largest population of foreign workers in Thailand and their number increased from 0.4 in 2001 to >1 million in 2009 [10]. In addition, there are significant numbers of illegal immigrants and displaced people with no nationality.

This study shows that *P. falciparum* and *P. vivax* transmission on the Thai–Myanmar border is seemingly influenced by different factors. The *P. vivax* case numbers and the proportion of *P. vivax* cases were strongly correlated with *An. minimus* and *An. maculatus* capture rates, indicating that a high level of local transmission might be causing these infections. Moreover, these vector species were positive with *P. vivax* by ELISA during the peak when they were abundant. In addition, *P. vivax* infections were approximately twice as likely to occur in the migrant populations (M1 and M2), compared to the Thai population (M0). This may partially be contributed to importation. However, there was no association of *P. vivax* infection with either the ‘recent’ (M2) or ‘longer-term’ (M1) migrant status. Thus, the difference in vivax infection rate between the migrant and the Thai populations is likely due to the differences in their living conditions, access to healthcare, use of malaria prevention measures and/or socio-economic factors. Migrants predominantly live in semi-permanent dwellings on the edge of the village, closer to potential breeding sites. In addition, the households are poorer, and ITN coverage and other precautions are also lower in this population [10, 19]. A significant negative age trend was observed in the probability to present with a *P. vivax* infection, suggesting of the acquisition of immunity in older populations.

In contrast, *P. falciparum* infections were not associated with the dynamics of the major vectors *An. minimus* and *An. maculatus*, but they were ~ four times more likely in recent migrants compared to the resident population. Also, there was no significant age trend in the probability to present with a *P. falciparum* infection as shown in previous studies in Southeast Asia [33, 34]. Further, fever was negatively associated with *P. falciparum* infection. *Plasmodium falciparum* case numbers fluctuated with agricultural seasonality, and more recent migrant (M2) patients presenting with infections were predominantly in the months of harvest season (November–December), which are characterized by an influx of labourers from Myanmar [35]. This suggests that importation may be a major cause of *P. falciparum* cases. It has been shown in similar settings on the Thai–Cambodian border that seasonal labourers are at a higher risk of being infected with vivax malaria [32, 35, 36]. Although, in this study, there was no association between the occupations specified by the patients and their malaria infection status and therefore occupations were not included into the final statistical model, other studies have shown that there are occupational risks of acquiring malaria infections in this region and on the Thai–Cambodian border, e.g., forest workers [32, 37].

A significant limitation of this study is that it was focused on only passively detected cases. However, the findings are in agreement with the results of a recent active case detection survey on a cohort of approximately 500 individuals in the same region, where ‘no citizenship’ (likely to be migrants) was identified as the most significant risk factor for asymptomatic malaria infections [19]. In this sense, active parasite detection should be conducted in order to quantify and characterize the asymptomatic parasite reservoir, since asymptomatic infections have been shown to represent an important source of transmission [12, 38–40]. Gametocyte carriage was not used as an explanatory variable in the models of this work as the gametocyte data were very scarce. However, future studies should include gametocytaemia as a factor for association with transmission.

The present study emphasizes the importance of mosquito surveillance. Several *An. minimus* mosquitoes were found positive for *P. vivax* and one *An. maculatus* mosquito was found positive for *P. falciparum*. Upscaled trapping studies may allow for the inclusion of sporozoite rates in the different mosquito populations into statistical models. Even though in this study *An. minimus* and *An. maculatus* constituted 85% of the captured vector population, other vectors, especially the deep forest vector *An. dirus*, should not be ignored as it has been shown to contribute significantly to transmission [10]. However, in the present study *An. dirus* represented <0.5% of the captured vector population as trapping was conducted inside the village. It is noteworthy that *An. minimus* and *An. maculatus* exhibit both anthropophilic and zoophilic biting behaviours. Therefore, fluctuation of cattle populations with the cattle trade may also affect the dynamics of these mosquito vectors [2]. The mobile cattle population may also cause a significant influx of infected mosquitoes. The contribution of these potentially ‘mosquito-imported’ infections should be further investigated through entomological studies.

## Conclusions

Entomological surveillance and its association with passive case detection data provided important insights into *P. falciparum* and *P. vivax* transmission on the Thai–Myanmar border. This study shows that *P. falciparum* infections were more concentrated in the recent migrant population and that the seasonality of *P. falciparum* cases mirrors that of agricultural activity. It can therefore be speculated that *P. falciparum* may be mostly imported. In contrast, *P. vivax* cases were significantly associated with the dynamics of the local mosquito population and less with migrant status, strongly suggesting local transmission. These findings suggest that *P. vivax* elimination may require considerably greater efforts than *P. falciparum* elimination and that sustained, well coordinated, border-transcending malaria control will be required to attain elimination status in this area and contain the expansion of drug resistance.

## Additional files

**Additional file 1: Figure S1.** Average number of mosquitoes monthly captured per trap.

**Additional file 2: Figure S2.** Number of *P. vivax* (A) and *P. falciparum* (B) cases per month reported by Suan Oi malaria clinic.

**Additional file 3: Table S1.** Patient characteristics for the M2 migrant population*. **Table S2.** Patient characteristics for the M1 migrant population*. **Table S3.** Patient characteristics for the Thai national population. **Table S4.** Occupation distribution among the different population strata.
