# Supplementary figures and images for: Imported Plasmodium falciparum and locally transmitted Plasmodium vivax: cross-border malaria transmission scenario in northwestern Thailand
Source: Malar J. 2017 Jun 21;16:258. doi: 10.1186/s12936-017-1900-2 (PMC5480133; doi:10.1186/s12936-017-1900-2)

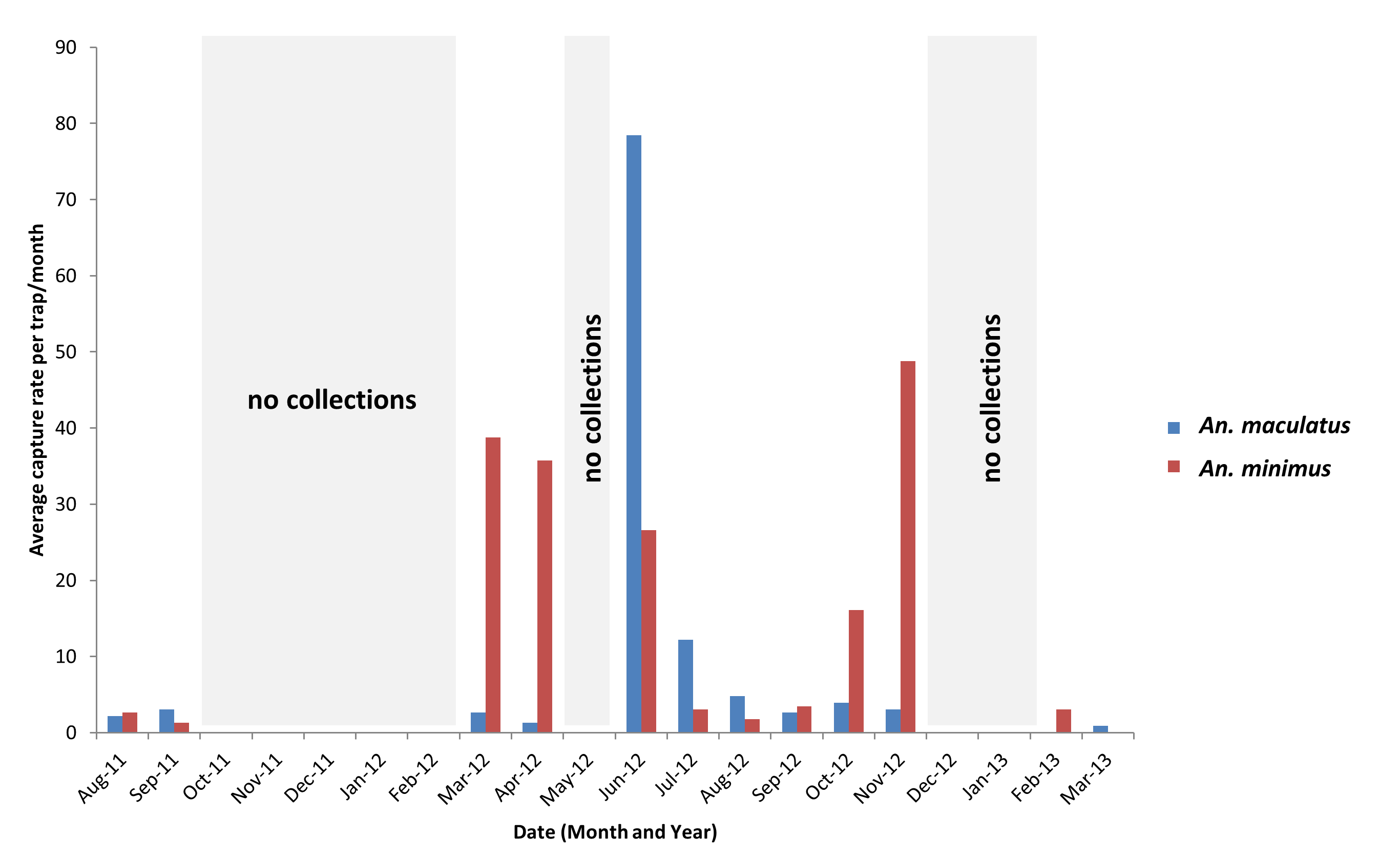

Supplement: Supplementary file 1 — Additional file 1: Figure S1. Average number of mosquitoes monthly captured per trap. [file 12936_2017_1900_MOESM1_ESM.tif]
