# Supplementary material for: Imported Plasmodium falciparum and locally transmitted Plasmodium vivax: cross-border malaria transmission scenario in northwestern Thailand
Source: Malar J. 2017 Jun 21;16:258. doi: 10.1186/s12936-017-1900-2 (PMC5480133; doi:10.1186/s12936-017-1900-2)
Supplement: Supplementary file 3 — Additional file 3: Table S1. Patient characteristics for the M2 migrant population*. Table S2. Patient characteristics for the M1 migrant population*. Table S3. Patient characteristics for the Thai national population. Table S4. Occupation distribution among the different population strata. [file 12936_2017_1900_MOESM3_ESM.pdf]

Supporting Information

Table S1: Patient characteristics for the M2 migrant population*.

Patient characteristics (n=522)	Number (%) or Mean (Range)
Age (years)	16.0 (0.25-79)
Temperature	36.96 (36.0-38.0)
Male	211 (40.4%)
Fever (>37.5^{o}C)	97 (18.5%)
**Malaria Infection**	
P. vivax	56 (10.7%)
P. falciparum	71 (13.6%)
P. vivax with fever	12 (21.4%)
P. falciparum with fever	8 (11.3%)

Note: M2 indicates migrants who have been in Thailand less than six months

Table S2: Patient characteristics for the M1 migrant population*.

Patient characteristics (n=1480)	Number (%) or Mean (Range)
Age (years)	19.4 (0-80)
Temperature	36.9 (36.0-37.9)
Male	732 (49.5%)
Fever (>37.5^{o}C)	640 (43.2%)
**Malaria Infection**	
P. vivax	163 (11.0%)
P. falciparum	84 (5.7%)
P. vivax with fever	72 (44.2%)
P. falciparum with fever	35 (41.6%)

Note: M1 are migrants who have been in Thailand for more than six months.

Table S3: Patient characteristics for the Thai national population.

Patient characteristics (n=2423)	Number (%) or Mean (Range)
Age (years)	20.4 (1-89)
Temperature	37.0 (34.5-38.0)
Male	1131 (47.6%)
Fever (>37.5^{o}C)	548 (38.5%)
**Malaria Infection**	
P. vivax	140 (5.8%)
P. falciparum	92 (3.8%)
P. vivax with fever	42 (30.0%)
P. falciparum with fever	20 (21.7%)

Table S4: Occupation distribution among the different population strata.

Occupation	M1	M2	Thai	Total
Unknown	284	29	447	760
Child	409	262	548	1219
Farmer/Rice Plantation	114	7	252	373
Forestry	0	0	3	3
Gardener	3	0	7	10
Public Service	0	0	16	16
Merchant	8	2	38	48
Cleric	6	17	3	26
Student	311	7	655	973
Military/Police	0	0	18	18
Disabled	1	0	1	2
Housewife	1	0	7	8
Labourer	314	196	387	897
Retired	29	2	37	68
Teacher	0	0	4	4
**Total**	**1480**	**522**	**2423**	**4425**
